# Supplementary material for: Hybrid BW-EDAS MCDM methodology for optimal industrial robot selection
Source: PLoS One. 2021 Feb 9;16(2):e0246738. doi: 10.1371/journal.pone.0246738 (PMC7872252; doi:10.1371/journal.pone.0246738)
Supplement: Attachment — Submitted filename: Response to Reviewers.docx [file pone.0246738.s001.docx]

**Point to point answers to the reviewers’ comments and suggestions**

Reviewer #1:

Thank you for inviting me as a reviewer for manuscript titled Hybrid BW-EDAS MCDM Methodology for Optimal Industrial Robot Selection; Journal PLOS ONE, Manuscript No: PONE-D-20-36145.
Presented methodology has potential in decision making field and I am giving support to the authors for investigation this topic. The strengths of this paper are: Relevant topic; Flow of the paper; and Explanation of the methods.

However, the author(s) need to consider the following points as limitation or further scope for refining the paper:

Thank you for the value able comments and suggestions to improve the manuscript. We have improved the manuscript according to your comments and suggestions.

Comment # 1: Need to better highlight the novelty of study in the introduction.

Answer: The novelty of the study is included and highlighted according to the suggestions.

Comment # 2: I suggest authors to clearly summarize what specific advantages brings your approach.

Answer: The advantage of this methodology is due to the consistent behavior of BWM, fewer calculation cost of EDAS method and a wide application are of both these methods. That is mentioned and highlighted in the paper.

Comment # 3: The authors didn’t explain why we need this research? What are motivations (Research Gaps) for this research? Clearly define motivations for your research.

Answer: Robot selection is a very big issue in this era due to large number of manufacturer and vast variety of robots in the market. So, there should be a user friendly and easy to understand methodology that can be adopted for this selection. The existing methods can have non consistent behavior or huge calculation cost that is rectified using this methodology. It is included and highlighted in the manuscript.

Comment # 4: What are limitations of existing approaches in the literature and can be eliminated by this study?
What are benefits of this approach?

Answer: Limitations of existing approaches are the non-consistency of pairwise comparisons for example in AHP comparisons can be non-consistent and the calculation cost of fuzzy ranking methods, for example triangular TOPSIS method. The proposed approach have following benefits, 1) weight deriving process is consistent, 2) it required fewer pairwise comparisons, 3) user friendly for DMs to provide opinions, 4) less calculation cost. It is included and highlighted in the paper.

Comment # 5: Literature review should be extended. Clearly define the gap based on literature review. In the literature review you should discuss why you have used BWM and not for example FUCOM, PIPRECIA, SWARA, LBWA etc. method. Some important references published in the last two years are missing. I suggest authors to read and add below references from the field:

[doi.org/10.31181/dmame2003078m](http://doi.org/10.31181/dmame2003078m);

[doi.org/10.31181/dmame2003019d](http://doi.org/10.31181/dmame2003019d);

[doi.org/10.31181/oresta2001072v](http://doi.org/10.31181/oresta2001072v);

[doi.org/10.31181/oresta1902001k](http://doi.org/10.31181/oresta1902001k);

[doi.org/10.31181/rme200101034c](http://doi.org/10.31181/rme200101034c).

Answer: Study gap is defined in introduction section and provided the benefits of use of BWM and reasons for not using other methods for example FUCOM, PIPRECIA, SWARA, LBWA etc. method. Important references are cited in the literature. It is included and highlighted in the paper.

Comment # 6: You should better organize sensitivity analysis section.

Answer: The sensitivity analysis is organized by improving the analysis and included in the paper.

Reviewer #2:

The paper is well structured.

Thank you for the value able comments and suggestions to improve the manuscript. We have improved the manuscript according to your comments and suggestions.

Comment # 1: The authors should better define motivations for their research.

Answer: Motivation of the paper is better defined and highlighted in the paper.

Comment # 2: Why you have used EDAS method?

Answer: EDAS method is used due to its simplicity, less calculation cost and huge application area.

Comment # 3: Why you have used BWM?

Answer: This methodology have following advantages, 1) weight deriving process is consistent, 2) it required fewer pairwise comparisons, 3) user friendly for DMs to provide opinions.

Comment # 4: Sensitivity analysis should be better organized.

Answer: The sensitivity analysis is organized by improving the analysis and included in the paper.

Comment # 5: Literature review section should be revised. Add more recent research papers from the filed.
Add more discussion on the results.

Answer: More discussion in introduction section is added and highlighted.

Comment # 6: Add managerial applications.

Answer: Managerial applications are included in paper as a section and highlighted.

Comment # 7: Add more future directions in conclusions.

Answer: Some future directions are added and highlighted.

Comment # 8: What are limitations of proposed methodology?

Answer: This method do not provide the process of suitable criteria selection among large number of criteria for the specific requirement. This method cannot handle fuzzy information.
